# Case report: Management challenges of late diagnosed 17‐alpha hydroxylase deficiency

**DOI:** 10.1002/ccr3.6962

**Published:** 2023-02-21

**Authors:** Dhoha Ben Salah, Oumeyma Trimeche, Mouna Elleuch, Wafa El abed, Ameni Salah, Fatma Abdelhadi, Hassen Kammoun, Wiem Feki, Zeineb Mnif, Khansa Chaabouni, Fatma Ayedi, Fatma Mnif, Nabila Rekik, Mouna Mnif, Nadia Charfi, Faten Hadj kacem, Mohamed Abid

**Affiliations:** ^1^ Department of Endocrinology Hedi Chaker Hospital Sfax Tunisia; ^2^ Department of Human Molecular Genetics, Faculty of Medicine Sfax Tunisia; ^3^ Department of Radiology Hedi Chaker Hospital Sfax Tunisia; ^4^ Department of Biochemistry Habib Bourguiba Hospital Sfax Tunisia

**Keywords:** 17‐alpha‐hydroxylase deficiency, congenital adrenal hyperplasia, disorders of sex development, hypertension, hypokalemia

## Abstract

Herein we report the intriguing case of a 42‐year‐old woman presenting with grade three hypertension, severe hypokalemia and primary amenorrhea, which revealed to be the complete form of 17 alphahydroxylase deficiency. We also discuss the challenging therapeutic approach as well as the outcomes and the follow‐up of this patient.

## Abstract 2

One should consider the diagnosis of 17 alpha hydroxylase as the etiology of hypertension, hypokalemia, and adrenal insufficiency regardless of the age of patients. Biochemical findings can be atypical with slightly elevated ACTH and normal aldosterone level. The management of this condition is challenging and should be multidisciplinary.

## 1 INTRODUCTION

The CYP17A1 gene encodes for the P450c17 enzyme which catalyzes two key enzymes: 17‐alpha hydroxylase and 17,20‐lyase. These enzymes are expressed mainly in the gonads and in the adrenal glands, and play a pivotal role in the biosynthesis of cortisol and sex steroids. The genetic defects of this gene result in a rare form of congenital adrenal hyperplasia (CAH). The main clinical determinants of this disorder are as follows: hypertension, hypokalemia, and disorders of sex development (DSD).^1^ Herein, we report the intriguing case of a 42‐year‐old woman presenting with grade three hypertension, severe hypokalemia, and primary amenorrhea, which revealed to be the complete form of 17 alpha‐hydroxylase deficiency (17 OHD). We also discuss the challenging therapeutic approach as well as the outcomes and the follow‐up of this patient.

## 2 CASE REPORT

Miss K.A. was assigned to our endocrinology department for the first time at the age of 42 years. She had a newly discovered hypertension associated with severe hypokalemia, which were found during her stay in the dermatology department for erysipelas. Otherwise, she had primary amenorrhea for which she never consulted. She also never reported an episode of hypokalemic paralysis. She is the offspring of a non‐consanguineous parents. As for her family history, we report neonatal death in two of her siblings and four of her cousins. Her brother had hypertension and type 2 diabetes.

**FIGURE 1:**
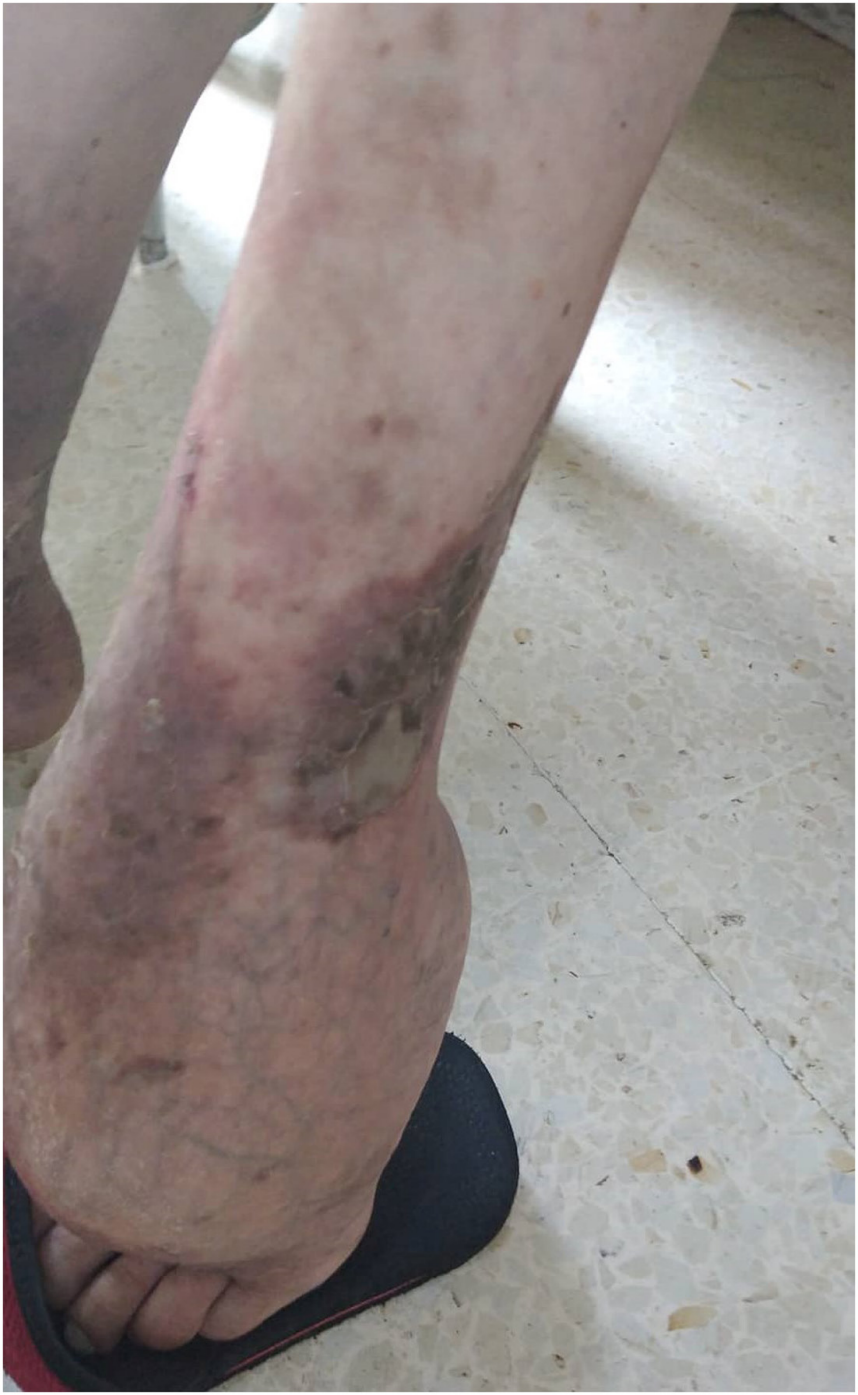
Left ankle malformation

On physical examination, she weighed 75 kg and her height was 1.82 m. She had non‐complicated grade 3 hypertension: 200/100 mmHg. Her neurological examination was abnormal as she had brisk reflexes of herknee tendon. The osteoarticular examination revealed irreducible flexum of her left elbow, reducible cubital deviation of her left hand and ankylosed left ankle (Figure 1). Regarding her gynecological examination, it showed feminine genitalia with no ambiguityand no hyperandrogenic features. Her Tanner score was therefore B1P1A1. Her EKG disclosed electric signs of hypokalemia: diffuse depressed ST segment and U waves. End organ complications of hypertension were evaluated and revealed the presence of left ventricle hypertrophy with slightly altered function.

Laboratory investigation revealed severe hypokalemia of 1.5 mmoL/L and metabolic alkalosis (ph, 7.54 and HCO3, 36.2 mmoL/L).

She exhibited a primary adrenal insufficiency as indicated by the extremely low cortisol level associated with moderately elevated ACTH. She also had high FSH and LH value with low estrogen and testosterone, implying a hypergonadotropic hypogonadism (Table 1). Pelvic MRI unveiled the absence of uterus, ovaries and the presence of two inguinal lesions resembling testicular structures. Her karyotype was 46, XY. Thus, we are confronting an XY, DSD with adrenal insufficiency combined with hypertension and hypokalemia. This association leads us to the diagnosis of a defect in the steroidogenesis pathway. Aiming topinpoint the exact level of the deficit, we conducted a series of hormonal measurements which showed high level of 11‐desowycorticosterone (DOC) and low levels of 17 hydroxy progesterone and androstenedione (Table 1). Putting all findings together, we can conclude that the patient had a complete form of 17OHD.

**TABLE 1:** Hormonal analyses in our case

	Value	Reference range
Cortisol (nmol/L)	15.17	
ACTH (pmol/L)	14.53	1.58–13.92
Aldosterone (nmol/L)	0.23	0.03–0.44
Renin (pmol/L)	<0.102	0.1–0.75
17 OHP (nmol/L)	0.76	0.39–1.54
DOC (pmol/ml)	3945	121.19–606
Androstenedione (nmol/L)	0.31	2.613.89–13.58
DHEAS (μmol/L)	0.19	1.08–5.89
FSH (UI/L)	73.5	1.5–12.4
LH (UI/L)	45.9	1.7–8.6
Estrogen (pmol/L)	<18.35	45.52–855.42
Testosterone (nmol/l)	0.03	2.85–8.01

*Abbreviation*: 17 OHP, 17 hydroxyprogesterone; ACTH, adrenocorticotropic hormone; DHEAS, dehydroepiandrosterone sulfate; FSH, follicle‐stimulating hormone; LH, luteinizing hormone.

The main differential diagnosis was the deficit in P 450 oxidoreductase deficiency (PORD) since our patient had skeletal malformations. However, the atypical deformities in her feet, the absence of craniostenosis, the mid face hypoplasia, and the radio humeral synostosis make this latter diagnosis unlikely. Similarly, the diminished level of the basal 17 hydroxy progesterone is inconsistent with this hypothesis. Deficiency of 11 beta hydroxylase could be discussed in this case given the association of hypertension, hypokalemia, and adrenal insufficiency in our patient. However, this hypothesis was dismissed given the absence of clinical signs of hyperandrogenic features in our patients and the non‐elevated levels of 17 OHP.

Given the patient's hypogonadal status, a bone mineral density was performed confirming the diagnosis of osteoporosis: *Z* score − 4.5.

Regarding her hypertension, we prescribed hydrocortisone (5 mg at 8:00 am, 5 mg at 12:00 am, and 10 mg at 12:00 pm). Nonetheless, this latter treatment was insufficient to obtain normal blood pressure (BP); thus, we added spironolactone, which also helps normalizes the kalemia of our patient. The evolution was marked by steady elevated BP, and therefore, we added Amlodipine and then Moxonide to our therapeutic arsenal. As for her hypokalemia, oral potassium supplementation along with Spironolactone was needed in order to attain normokalemia. One month after therapy, her blood pressure was 120/80 mmHg and she had normal potassium level of 4.6 mmoL/L. Echocardiography showed an improvement of her left ventricular function.

## 3 DISCUSSION

Congenital adrenal hyperplasia (CAH) encompasses different genetic anomalies. 17 OHD represents approximately 1% of all causes of CAH, which is inherited in an autosomal recessive pattern. The lack of this enzyme results in an excess of potent mineralocorticoids such as DOC, which leads to hypertension and hypokalemia. Additionally, 17 OHD causes a deficit in the cortisol production and thus an adrenal insufficiency. Regarding the gonadal sex steroids, 17 OHD precipitates their decrease and therefore generates a hypergonadotropic hypogonadism.^2^ Depending on the severity of the molecular abnormality, various forms of this disease are reported. While the complete enzymatic deficiency results in a severe phenotype with an absence of virilization in 46, XY patients and impuberism in 46, XX, which corresponds to the phenotypic presentation of our patient, the partial form is characterized by a milder clinical manifestation: more frequently encountered normotension, secondary amenorrhea and possible virilization in 46, XYmales.^3^ Biochemically, 17 OHD is characterized by high levels of DOC, and low levels of DHEAS, androstenedione, 17 OHP, and cortisol.^2^

The management of 17 OHD is based mainly on the treatment of hypertension, hypokalemia, and hypogonadism. The cornerstone treatment is hydrocortisone which enables the reinstitution of the negative feedback on the corticotropic axis and thus diminishes ACTH levels and consequently the production of the excess mineralocorticoids. This helps to control hypertension and hypokalemia. Some cases of complete reversibility of hypertension and hypokalemia were reported in cases of CAH due to 11 beta hydroxylase using corticosteroids but not with 17 alpha hydroxylase, to our knowledge.^4^, ^5^ Besides, spironolactone acts both as an antihypertensive agent and a potassium sparing diuretic, is considered a drug of choice in these cases. As for the management of hypogonadism, 46, XY patients with female phenotype can be prescribed with hormonal therapy aiming to prevent osteoporosis, cardiovascular complications of sex steroids deficiency but also to induce secondary sexual characteristics.^6^ Furthermore, the presence of the Y material and specifically the TSPY: testis specific protein y linked 1 gene, is incriminating in the development of gonadal malignancies. While gonadectomy is not recommended in 17 OHD patients due to lack of data concerning this subject, a radiological surveillance of the testis seems crucial in order to detect neoplasm.^7^

Our case has many peculiarities. First, the  advanced age of diagnosis of 17 OHD. Indeed, most of the 17 OHD cases are diagnosed at puberty, and the primary clinical manifestation of this disorder is usually the abnormal sexual development. Interestingly, patients with 17 OHD never exhibit signs of adrenal crisis. This can be explained mainly by the excess of corticosterone, an agonist hormone of the glucocorticoid receptor. Consequently, this phenomenon helps to clarify the late age of the diagnosis.

Secondly, the moderate elevation of ACTH level seen in our patient could result from the increasing level of corticosterone which can impact the ACTH secretion and explain the absence of higher levels of this latter hormone in some cases.^8^

Another intriguing finding of our case is the normal level of aldosterone. Patients with 17 OHD generally exhibit low aldosterone concentration, mainly consequential of the suppression of the renin angiotensin system by the elevated DOC. We suggest that the conjunction of this latter effect with the conversion of corticosterone to aldosterone by a secondary pathway: Corticosterone methyl oxidase resulted in the normal level of aldosterone.^9^

Finally, the skeletal deformations found in our patient which makes our case unique. In fact, no other cases of 17 OHD showed similar features. This leads us to the differential diagnosis: P450 oxidoreductase deficiency. However, the atypical skeletal malformations, the normality of the 17 OHP pleads against this latter diagnosis.^10^ Nevertheless, a predominant deficit in 17‐alpha hydroxylase over the 21 hydroxylase seen in some patients of PORD can be consisting with our case.

Regarding the limitations of this study, we recognize that the genetics are a crucial substance for the diagnosis of this condition as well as the hormonal evaluation of 17 OHP, progesterone, and pregnenolone before and after ACTH stimulation test. Unfortunately, these examinations are not available in our hospital.

## 4 CONCLUSION

This report emphasizes the importance of exploring pubertal delay and the need for a precocious diagnosis of 17‐alpha hydroxylase deficiency as it can alleviate the complications of this disease notably hypertension and hypokalemia.

## AUTHOR CONTRIBUTIONS

Dhoha Ben salah provided the idea of the case report and patient enrolment. Trimeche Oumeyma drafted the manuscript. Elleuch. Mouna, El abed. Wafa, Salah. Ameni, Abdelhadi. Fatma, Kammoun. Hassen Feki. Wiem, Mnif. Zeyneb, Chaabouni. K, Ayedi. Fatma, Mnif. Fatma, Rekik. Nabila Mnif. Mouna, Charfi. Nadia, and Hadj kacem Faten were involved in patient enrolment and revising of the manuscript. Abid Mohammed approved the manuscript.

## CONFLICTS OF INTEREST

All authors do not report any conflicts of interest.

## CONSENT

A written and oral informed consent was obtained from the patient.

## Data Availability

Data sharing is not applicable to this article as no datasets were generated or analyzed during the current study.
